# Reference percentiles for bioimpedance body composition parameters of healthy individuals: A cross-sectional study

**DOI:** 10.1016/j.clinsp.2022.100078

**Published:** 2022-09-07

**Authors:** Marina Azambuja Amaral, Eduardo Mundstock, Camila H. Scarpatto, Wilson Cañon-Montañez, Rita Mattiello

**Affiliations:** a Pontifícia Universidade Católica do Rio Grande do Sul, Porto Alegre, RS, Brazil; b Centro Universitário Ritter dos Reis (UniRitter), Porto Alegre, RS, Brazil; c Secretaria da Educação, Esporte e Lazer de Canela, Canela, RS, Brazil; d Universidad de Antioquia, Medellín, Antioquia, Colombia; e Universidade Federal do Rio Grande do Sul, Porto Alegre, RS, Brazil

**Keywords:** Body composition, Body fat, Bioelectrical impedance analysis, Fat-free mass, Reference standard

## Abstract

**Objectives:**

The aim of this study was to estimate the percentage distribution of body composition parameters for healthy people at different ages from the assessment of electrical bioimpedance.

**Methods:**

A cross-sectional study of healthy Brazilian aged 5 years and older. Were evaluated: total body fat; percent body fat; fat-free mass; percent lean mass; fat mass index; and fat-free mass index.

**Results:**

Of 1240 participants, with a median age of 27.0 years, 52.5% were female, and 73.7% were Caucasian. Most of the body composition variables were associated with age. The fat-free mass increased from youth to adult and decreased in the elderly in both sexes, with higher values in males than in females. In males, the percentage of lean mass has higher values in adolescence compared to childhood, and in adults compared to the elderly, when analyzed from the 50^th^ percentile. In women, fat-free mass compared to adulthood, values were higher in childhood and lower in older ages.

**Conclusions:**

The study is the first to describe the Brazilian reference values for most clinical parameters of bioimpedance in percentiles stratified by different life cycles and sex. These findings can be very useful in clinical practice for health promotion and monitoring the nutritional status of the individual.

## Highlights

• Body composition is considered an important predictor in different clinical and health promotion scenarios.
• This is the first study to describe the reference values of body composition composed of a sample of Brazilians stratified by different life cycles, sex, and race.
• This is the first study that includes the evaluation of all parameters of body composition assessed from electrical bioimpedance.

## Background

Body composition parameters can be useful prognostic factors for morbidity^1^ and mortality. The lower lean mass has been considered a good prognostic factor predicting mortality in patients with chronic obstructive pulmonary disease,^2^ sarcopenia,^3^ and cancer.^4^ Conversely, the excess Fat Mass (FM) above the recommended age is a predictor of the development and severity of the disease in different medical conditions, such as obesity,^5^ nonalcoholic fatty liver disease,^6^ diabetes mellitus,^7^ dyslipidemia,^6^ hypertension,^5^ and cancer.^8^

According to the latest guideline for the treatment of obesity 2020, the assessment of body adiposity is essential given all the complications that excess Body Fat (BF) brings to individuals' health.^9^ Among the body composition assessment methods, electrical bioimpedance has been the most feasible method for studying body composition in both clinical practice and the research field,^10^ as it is a method with adequate precision, non-invasive, easy to apply, safe, and relatively inexpensive.

Despite this measurement method's applicability, the reference values generated from individuals with a wide age range and generalizable eligibility criteria for the different electrical bioimpedance parameters are scarce.^11^, ^12^, ^13^, ^14^, ^15^ Population-specific body composition reference data help to elucidate age-related changes associated with health problems. They will allow more accurate characterization of individuals at the most significant risk of disability and morbidity so they can receive targeted support.^16^^,^^17^

Among the evidence that suggests reference values for body composition parameters evaluated by electrical bioimpedance, most studies evaluate only specific age range as children,^5^ children and adolescents,^12^^,^^13^^,^^18^, ^19^, ^20^, ^21^, ^22^, ^23^, ^24^ adults,^25^ and the elderly^11^^,^^14^^,^^15^^,^^26^, ^27^, ^28^, ^29^ or just the elderly.^30^^,^^31^ These studies were carried out with specific demographic characteristics in Germany,^18^ China,^21^^,^^26^ Colombia,^19^ United States,^22^^,^^24^^,^^28^ Western Europe,^11^ Great Britain,^14^^,^^28^ India, ^23^ England,^13^ Japan,^20^^,^^30^ Sweden,^31^ Switzerland^15^^,^^27^ and Turkey.^12^ The body composition parameters presented in the published studies are Body Fat percentage (%BF), Fat Mass (FM) in Kg, Lean Mass (LM) in Kg, Fat Mass Index (FMI), and Fat-Free Mass Index (FFMI).^11^, ^12^, ^13^, ^14^, ^15^^,^^18^, ^19^, ^20^, ^21^, ^22^, ^23^^,^^25^, ^26^, ^27^, ^28^, ^29^, ^30^, ^31^

The inhomogeneous nature of the various body compartments and large variations in cross-sectional areas are likely responsible for the lack of portability of electrical bioimpedance references values from one population to another.^11^ In Brazil, only one study presented reference values of the percentage of fat with adult individuals aged 18 to 39 years in the southern region using the bioelectrical impedance method. However, without showing the sample's socio-cultural and economic aspects and other bioimpedance parameters.^25^ Therefore, the present study aimed to estimate the percentage distribution of FFM, FMI, and FFMI for healthy individuals of both sexes at different ages and ethnicities from electrical bioimpedance.

## Methods

### Study design

This cross-sectional study followed the guidelines for reporting observational studies – STROBE Statement.^32^

### Study population

Healthy community-dwelling individuals aged 5 years and older, of both sexes, were invited to participate in the study. Exclusion criteria were contraindications to electrical bioimpedance, such as diseases affecting the skin's electrical resistance, pregnancy, persons with an implanted pacemaker or cardioverter-defibrillator, and persons with amputation or using prosthesis/orthosis. Participants were considered healthy if they had not been diagnosed with any chronic illness or used medication for chronic diseases continuously.

Data were collected in companies, and at events in cities in southern Brazil. Recruitment occurred through word of mouth.

### Data measurements

Sociodemographic variables were obtained through structured interviews. These included age (years), sex (male or female), race (categorized into white, black, or others – brown, Asian, and indigenous were grouped together to homogenize the size of the categories), and a total of family monthly income. Age was described in years and categorized into life cycles: children (age 5 to 9 years); adolescents (age 10 to 19 years); adults (age 20 to 59 years); and older adults (≥ 60 years of age).^33^

Body mass was measured with the participant in the standing position, with the least possible clothing and no shoes, using a calibrated digital scale (Charder MS6121). Height was measured with the participant standing barefoot with parallel feet and heels together, arms along the body, and head in the Frankfurt plane, using a Sanny compact stadiometer and a tape measure to the nearest 0.1 cm (American Medical do Brasil Ltda, São Bernardo do Campo, SP, Brazil).

Bioimpedance Multi-frequency InBodyS10 (Ottoboni, Rio de Janeiro, RJ, Brazil) was used to assess the body composition parameters. The InBodyS10 showed excellent agreements with Dual-Energy X-Ray Absorptiometry (DEXA) regarding to whole body LM, FM, and %BF.^34^ The applied current was 100 μA (1 kHz) and 500 μA and the frequency was 50 kHz. The hand electrodes were attached to each thumb and middle finger, while the foot electrodes were positioned between the anklebone and the heel, covering as much area as possible. The Bioelectrical Impedance Analysis (BIA) was performed with the participants on a nonconductive surface in the standing position, with their legs apart and arms held away from their body and wearing the least amount of clothing possible and no metal jewelry. The standard guidelines were followed to instruct regarding the fasting state of the subjects before the BIA.^35^ All measurements were performed by one of the four experienced researchers according to the manufacturer's instructions using a standardized technique. All the participants completed three evaluations, and the average of the three values was considered as their result. The following body composition parameters were assessed: total BF (kg); percent BF (%); fat-free mass (FFM) (kg); percent LM (%); FMI (kg/m²); and FFMI (kg/m²). The FMI was calculated by the ratio between the FM (in kg) and the height (in meters) squared, and the FFMI was calculated by the ratio between the FFM (in kg), and the height squared.

The researchers received previous training to standardize the application of the questionnaire, anthropometric measurements, and electrical bioimpedance. All examinations were performed indoors, and the patients were advised that they should have 3 hours of fasting before the test.

### Statistical analysis

#### Sample size

The Statistical Power Analyses G*Power program was used to estimate the sample size. Considering a regression determination coefficient of 0.1 (small effect size) of body composition parameters and age range, a power of 95%, and a significance level of 0.05, the minimum number of participants necessary was 124 subjects. However, the authors also consider the minimum number of 50 participants for each sex and life cycle.

#### Data analysis

Data were expressed as mean (SD) or median and interquartile range (IQR, 25^th^‒75^th^ percentiles) for continuous variables and absolute and relative frequencies for categorical variables. The relationships between outcome variables (body composition variables) and the predictor variable (age) were investigated using quantile regression analysis (5^th^ to 95^th^ quantiles), stratified by sex. Quantile regression methods allow for estimation of differing relationships at different parts of the distribution of the dependent variables (body composition parameters). All tests were 2-sided, and p-values <0.05 were considered statistically significant. Statistical analyses were performed with SAS version 9.4 (SAS Institute Inc., Cary, NC, USA).

#### Ethical aspects

The present study was conducted according to the Declaration of Helsinki^36^ and was approved by the Ethics Committee of the Pontificia Universidade Católica do Rio Grande do Sul, Brazil, under approval number 2.187.802. All adult participants provided written informed consent. For underage participants, consent was obtained from their parents or guardians. An assent form was read and explained to the children/adolescents, and their signature was then collected.

## Results

A total of 1240 participants aged 5 years and older were evaluated. Of these, 651 (52.5%) were female and 777 (73.7%) were Caucasian, 116 (11.0%) were African American and 161 (15.3%) had other ethnicities (Table 1). The mean (SD) age of the participants was 7.4 (1.4) years for children, and 13.6 (2.7) years for adolescents. The median monthly family income was US$ 931.00 (IQR, US$ 468.00‒2128.00) 8 (10.2) years for adults and 71.4 (10.2) years for older adults (Table 1).

**Table 1:** Socio-demographic distribution of the sample.

	**n (%)**
**Sex**	
Women	651 (52.5)
Men	589 (47.5)
**Ethnicity**	
Caucasian	777 (73.7)
African American	116 (11.0)
Other	161 (15.3)
**Family monthly income**	
< U$ 468.00	232 (25.2)
U$ 469.00 to U$ 936.00	235 (25.5)
U$ 937.00 to U$ 1,995.00	204 (22.1)
> U$ 1995.00	251 (27.2)
**Age**	
5 to 9 years	137 (11.3)
10 to 19 years	288 (23.8)
20 to 59 years	716 (59.2)
≥ 60 years	67 (5.7)

Table 2 shows the distribution of body composition parameters by sex and age in the 5^th^, 10^th^, 25^th^, 50^th^, 75^th^, 90^th^, and 95^th^ percentiles. Median body composition data and variations adjusted for the life cycle and both sexes are described in Table 3. All body composition parameters are presented in 25^th^ and 75^th^ percentile according to life cycle and sex. Most body composition variables were associated with age. The only lean mass percentage at the 25^th^ percentile in males was not significantly associated with age.

**Table 2:** Body composition sample measures by sex and age.

	**n**	**Mean±SD**	**Percentile**						
			**5^th^**	**10^th^**	**25^th^**	**50^th^**	**75^th^**	**90^th^**	**95^th^**
**Total body fat (kg)**									
Men									
5 to 9 years	58	7.5±5.3	2.3	2.4	3.6	5.9	9.6	17	19.2
10 to 19 years	134	10.6±9.6	2.4	3.2	5.3	7.7	12.2	21.3	27.5
20 to 59 years	371	18.1±8.9	7	8.7	11.9	16.5	22.4	28.7	35.5
≥60 years	16	22.2±8.0	12.3	14	15.2	21.3	27.4	32.2	32.2
**Women**									
5 to 9 years	79	7.1±5.1	2.2	2.6	4	5.4	8.3	15.4	17.8
10 to 19 years	154	16.0±9.1	6.2	7.6	9.8	13.4	21.4	25.2	35.5
20 to 59 years	345	22.9±11.0	10.1	11.2	14.4	20.4	28.5	38.5	43.3
≥60 years	51	25.3±9.5	12.9	15	19.9	24.6	29.4	36.7	47.2
**Percentage of body fat (%)**									
**Men**									
5 to 9 years	58	21.4±10.0	9.3	10	14.4	19	28.1	36.4	40
10 to 19 years	134	18.1±10.2	4.4	7.4	12	15.5	22.8	32.4	35.7
20 to 59 years	371	21.11±7.7	10.2	12	15.5	20.2	25.8	31.1	34.5
≥60 years	16	27.8±8.8	18.1	15	20.7	27.6	33.8	38.1	38.1
**Women**									
5 to 9 years	79	22.6±10.2	8.6	11.2	15.9	21	25.7	38.2	43.5
10 to 19 years	154	27.5±8.8	15.6	18.3	21.8	26	33.3	41.1	43
20 to 59 years	345	32.4±9.2	18.5	20.8	25	32.3	38.8	44.3	48
≥60 years	51	38.2±9.0	24.4	28.4	33.7	39.4	43.4	49.2	50.7
**Fat free mass (kg)**									
**Men**									
5 to 9 years	58	24.3±5.0	19.2	19.7	21.3	24.3	26.5	28.4	29.4
10 to 19 years	134	45.5±15.3	33.5	35.4	39.2	44.6	50.3	56.3	60.6
20 to 59 years	371	64.9±8.6	51.2	54.7	59.2	64.3	70.3	75.9	79.1
≥60 years	16	56.8±10.5	44.8	44.8	47.2	53.7	66	66.2	77.1
**Women**									
5 to 9 years	79	21.7±4.3	16.3	17.5	19.5	21.3	23.8	26.3	27.6
10 to 19 years	154	38.7±7.9	28.9	30.6	33.5	38.2	42.7	48.3	51.6
20 to 59 years	345	44.5±5.4	36.3	39.1	40.7	44.1	47.6	51.2	53.1
≥60 years	51	39.8±7.5	32	33.5	36.7	38.5	42.4	45.9	48.8
**Percentage of lean mass (%)**									
**Men**									
5 to 9 years	58	73.8±9.6	56.5	59.5	67.7	76	80.8	84.6	85.6
10 to 19 years	134	77.0±9.6	60.3	63.4	72.7	79.1	83	87	90
20 to 59 years	371	74.4±7.2	61.8	64.9	69.9	75.2	79.4	83.1	84.8
≥60 years	16	68.0±8.3	58.3	62.2	68.5	74.5	75.6	75.6	77.2
**Women**									
5 to 9 years	79	72.9±9.6	53.2	58.4	69.4	74.4	79.3	83.7	86.5
10 to 19 years	154	68.1±8.3	53.6	55.3	63	69.4	73.6	76.7	79.8
20 to 59 years	345	63.6±8.5	49.2	52.4	57.5	63.8	70.5	74.5	76.7
≥60 years	51	58.0±8.1	46.5	48.2	52.6	56.9	62.1	67.3	70.9
**Fat mass index (kg/m^2^)**									
**Men**									
5 to 9 years	58	4.2±2.8	1.3	1.5	2.2	3.3	5.8	8.1	10
10 to 19 years	134	4.1±3.7	0.8	1.2	2.1	2.9	4.8	8.8	10.2
20 to 59 years	371	5.9±2.9	2.2	2.8	3.8	5.3	7.3	9.6	10.8
≥60 years	16	7.8±3.2	4.2	4.9	5.4	7.7	9.8	11.5	11.5
**Women**									
5 to 9 years	79	4.3±3.0	1.2	1.7	2.4	3.4	4.7	9.5	10.9
10 to 19 years	154	6.4±3.4	2.8	3.2	4.1	5.4	8.2	10.7	14.6
20 to 59 years	345	8.9±4.4	3.7	4.3	5.6	7.9	11.4	15	17.1
≥60 years	51	10.7±4.0	5.4	6.4	7.9	10.4	13	16.6	19.3
**Fat free mass index (kg/m^2^)**									
**Men**									
5 to 9 years	58	14.2±0.9	12.7	12.9	13.5	14.2	15	15.2	15.4
10 to 19 years	134	17.1±2.8	14.5	15	15.9	16.8	18.1	19.4	20.5
20 to 59 years	372	20.8±2.2	17.1	18.3	19.5	20.8	22.3	23.6	24.1
≥60 years	16	19.7±2.0	16.5	16.5	18.2	20.2	21.2	22	23.2
**Women**									
5 to 9 years	79	13.4±0.9	11.6	12.2	12.9	13.4	13.9	14.5	15
10 to 19 years	154	15.6±1.6	13.3	13.7	14.5	15.5	16.6	17.8	18.4
20 to 59 years	345	17.1±1.7	14.8	15.3	16	16.9	18	19.5	20.3
≥60 years	51	16.6±2.3	14.3	14.6	15.4	16.5	17.5	18.1	19.8

**Table 3:** Estimated effects on body composition parameters with age at 25^th^ (young), 50^th^ (adult), and 75^th^ (old age) percentiles.

**Body composition parameters**	**Age**	**Men**				**Women**			
		**95% CI Estimates**	**Lower**	**Upper**	**p**	**95% CI Estimates**	**Lower**	**Upper**	**p**
Total body fat (kg)	P25	0.24	0.21	0.28	<0.0001	0.25	0.22	0.28	<0.0001
	P50	0.31	0.26	0.36	<0.0001	0.27	0.22	0.32	<0.0001
	P75	0.29	0.21	0.36	<0.0001	0.28	0.12	0.43	0.0003
Percentage of body fat (%)	P25	0.17	0.11	0.22	<0.0001	0.23	0.18	0.28	<0.0001
	P50	0.16	0.12	0.21	<0.0001	0.26	0.22	0.30	<0.0001
	P75	0.07	0.01	0.12	0.0127	0.19	0.13	0.25	<0.0001
Fat free mass (kg)	P25	0.75	0.68	0.82	<0.0001	0.25	0.19	0.30	<0.0001
	P50	0.53	0.38	0.67	<0.0001	0.19	0.13	0.24	<0.0001
	P75	0.39	0.25	0.52	<0.0001	0.19	0.11	0.27	<0.0001
Percentage of lean mass (%)	P25	-0.06	-0.12	0.0015	0.05	-0.17	-0.22	-0.13	<0.0001
	P50	-0.14	-0.18	-0.10	<0.0001	-0.24	-0.28	-0.20	<0.0001
	P75	-0.15	-0.19	-0.11	<0.0001	-0.21	-0.26	-0.16	<0.0001
Fat mass index (kg/m²)	P25	0.06	0.04	0.07	<0.0001	0.08	0.06	0.09	<0.0001
	P50	0.08	0.06	0.09	<0.0001	0.10	0.08	0.12	<0.0001
	P75	0.05	0.03	0.07	<0.0001	0.09	0.05	0.14	<0.0001
Fat free mass index (kg/m²)	P25	0.12	0.11	0.14	<0.0001	0.05	0.04	0.06	<0.0001
	P50	0.12	0.09	0.15	<0.0001	0.05	0.04	0.07	<0.0001
	P75	0.09	0.06	0.13	<0.0001	0.06	0.04	0.08	<0.0001

**Fig. 1:**
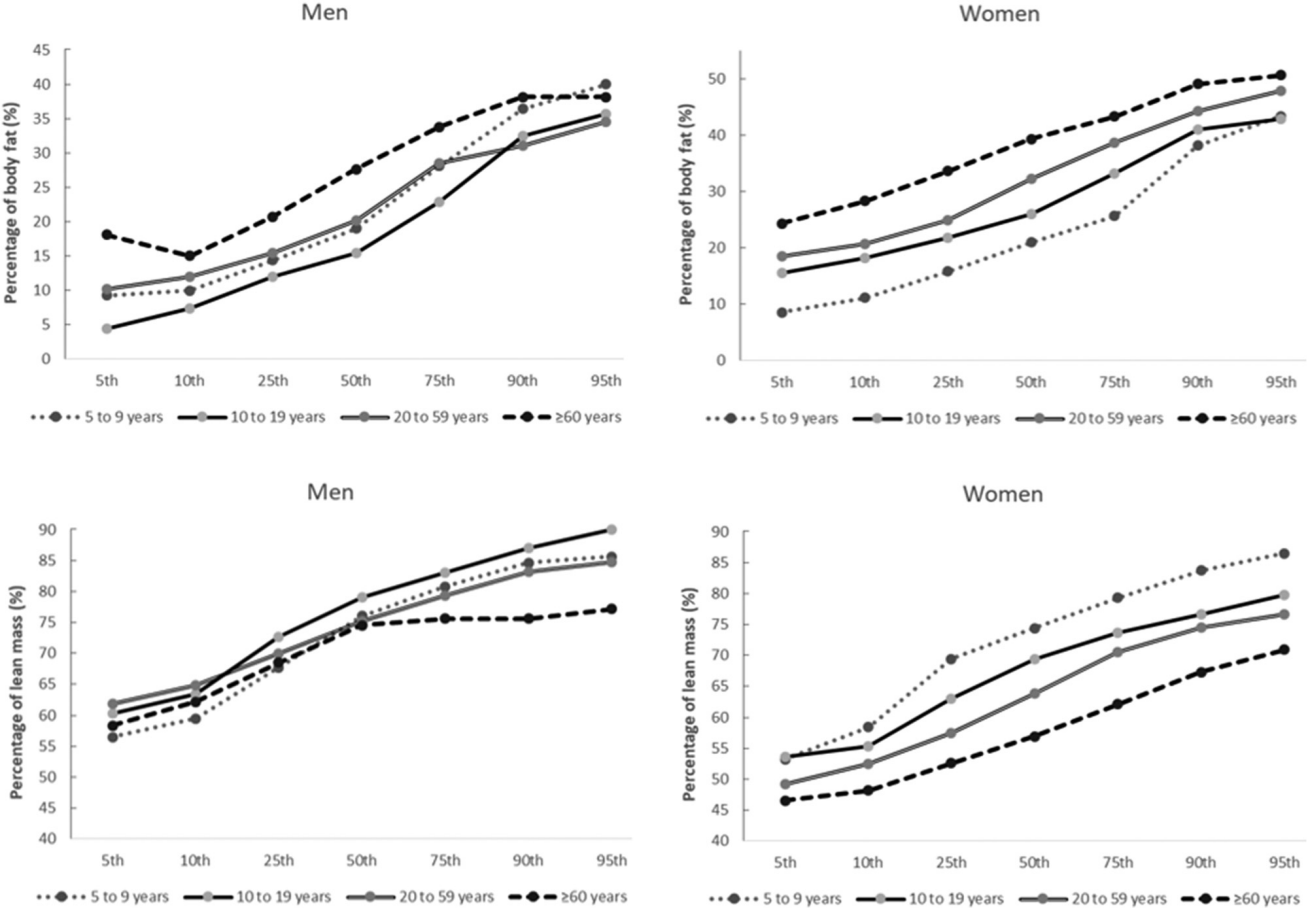
Percentage of body fat and lean mass parameters by sex life cycles.

**Fig. 2:**
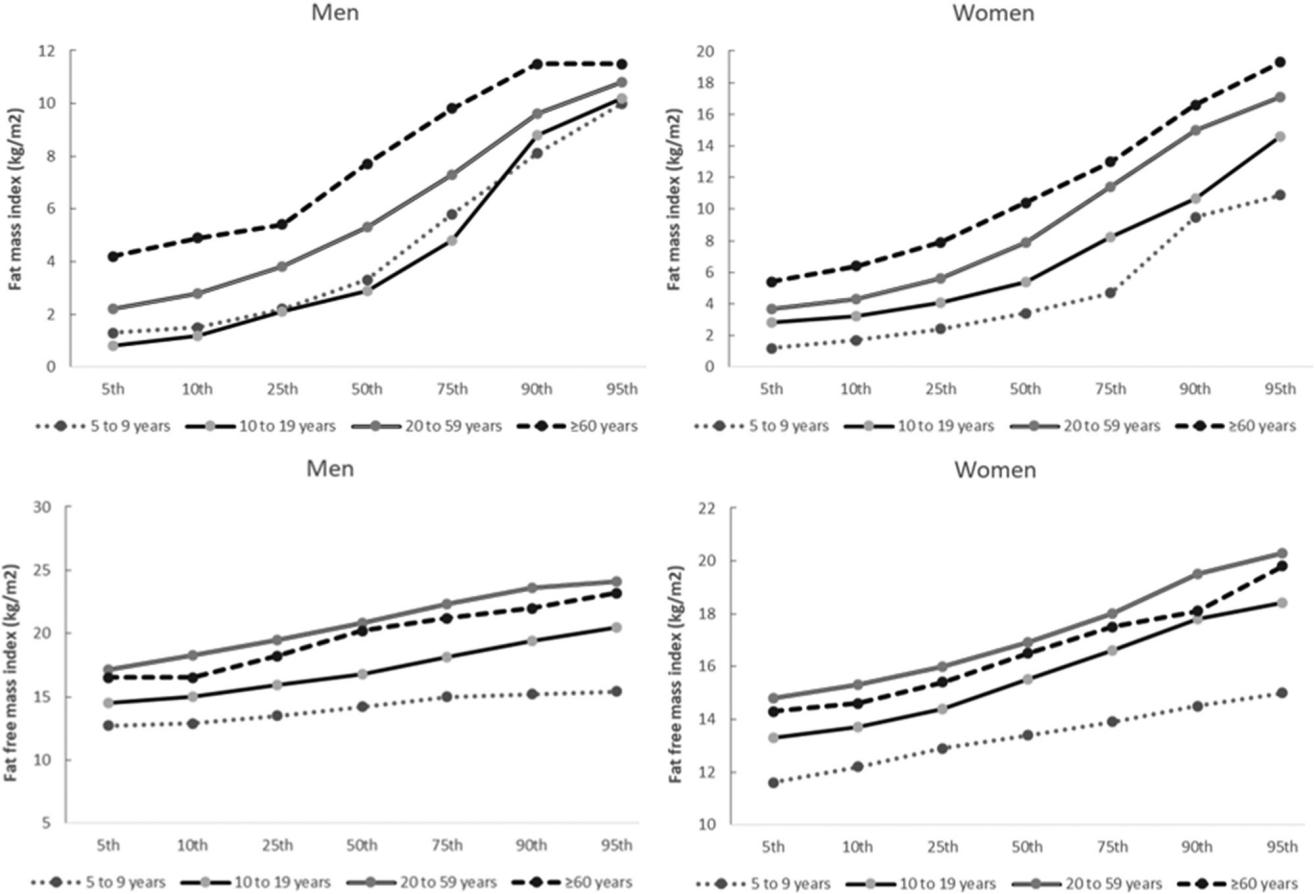
Fat mass index and fat free mass index parameters by sex and life cycle.

Figures 1 and 2 show body composition parameters for men and women according to the 5^th^, 10^th^, 25^th^, 50^th^, 75^th^, 90^th^, and 95^th^ percentiles in each life cycle (Children, Adolescents, Adults, and Seniors).

The figures show a trend towards higher %BF and FMI in aging. The FFMI showed a tendency to be higher in childhood and adulthood and then to be lower in older adults. Even though the data show a significant increase in body fat with age in males, the effect of growth is not significant since confidence intervals overlap between different age groups.

## Discussion

The present study estimated the percentile distribution of body composition parameters, as measured by electrical bioimpedance, for a healthy population including different ethnicities, stratified by sex, and adjusted for the life cycle. Age was associated with all body composition parameters at most percentiles in both sexes.

Changes in body composition with age may result from a combination of age and sex-related physiological changes.^37^ The behavioral differences in the body composition parameters between men and women are more evident in childhood and adolescence. Most studies show that, in females, the %BF is lower in childhood and the elderly; and higher in adolescence^12^^,^^13^^,^^19^ and adulthood. However, in males, it is lower in adolescence compared to childhood and higher from the age of 20 to the elderly. In females, the FFM peak is childhood,^12^^,^^18^^,^^19^^,^^23^^,^^25^ and in males, it occurs in adolescence. These sex differences may be due to increased longitudinal growth during puberty in males, while in females, there are increased energy reserves due to sexual maturation.^38^ At puberty, a series of physiological changes occur, including hormonal fluctuations and body composition changes, which accentuate sexual dimorphism. There is a change in the proportion of the body, with boys gaining more FFM and assuming an android form, while girls continue to accumulate more FM and assume a gynecoid form.^39^ The trend of growth in body parameters is evident in all variables and age groups. For the percentage of lean mass, there is a smaller difference between the percentages for thin or average people, but for elderly people at the upper weight limit, the percentage of lean mass has a tendency to be lower compared to young and adult people at the upper weight limit.

FFMI and FMI have been proposed as possible replacements for body mass index, as they consider the distribution of body composition and not just the absolute value of body mass. In women, the FMI is lower in childhood and higher in adolescence. However, in males, while the data of this study show that the FMI is higher in childhood and lower in adolescence, a similar study shows a trend towards an increase in this rate from children to adolescents.^15^ Regarding the FFMI, the values found are similar to previous work, showing that women have higher values compared to men,^13^, ^14^, ^15^^,^^22^^,^^23^^,^^27^^,^^30^ presenting a peak in adulthood,^13^^,^^22^^,^^23^ with a slight tendency to be smaller in aging.^11^^,^^13^^,^^22^^,^^23^^,^^30^

Thus, the importance of body composition as a determinant of health and prognosis is recognized, being of considerable interest in the evaluation of clinical and nutritional status in epidemiological, clinical, and scientific settings. Additionally, the quantile regression analysis performed in the present study allowed us to observe the percentile distribution of %BF according to life cycle and sex, rather than only body composition values categorized according to the reference values currently used in the assessment of body composition as a determinant of risk and health.^11^^,^^13^, ^14^, ^15^^,^^22^, ^23^, ^24^ Although DEXA is considered the gold standard, it emits a small dose of radiation. In addition, it is expensive equipment, which makes it unfeasible to be widely used.

This study is not free of limitations. Dual-energy X-Ray absorptiometry or body plethysmography were not used, which are considered the gold standard to evaluate body composition.^24^ Nevertheless, this study used electrical bioimpedance, making it clinically relevant since electrical bioimpedance equipment is widely used in clinical practice, with great potential to be used individually and in large populations. A recently published study validated the FFM prediction equation, using DEXA data evaluated by and confirmed the possibility of electrical bioimpedance to predict body composition in this population.^40^ Besides, DEXA emits a small dose of radiation, which can be considered an adverse effect of the method. Another limitation of the study was that the authors did not present the results by race. However, when the authors compare the distribution of the present sample, the authors realized that it is similar to Brazil. Despite the differences in the distribution of age groups, the uncertainties related to these data were expressed in confidence intervals.

The present study did not assess food consumption. However, this is a complete part of the nutritional assessment but not essential for evaluating the body composition. The authors have not included a probabilistic sample of the Brazilian population. However, the strength of this study is referenced BIA parameters in a healthy population covering all life cycles, thereby allowing the reference values to be used more comprehensively in the clinical practice for populations of mixed ethnicities.

## Conclusions

The present study estimated most clinical parameters of bioimpedance in percentiles including different ethnicities, stratified by different life cycles and sex, by applying the quantile regression model technique of a sample of Brazilians. Age was associated with all body composition parameters at most percentiles in both sexes, showing a trend of increasing fat mass with age with no significant difference between age groups, while lean mass showed a tendency to be higher in childhood and adulthood and then to be lower in older adults. The determination of body composition is important in clinical practice and in the evaluation of populations since there is a direct association between both high body fat and low lean mass with several metabolic changes. Thus, the mapping of population characteristics are subsidies for the development of strategies for health promotion and prevention of chronic non-communicable diseases.

## Authors’ contributions

Marina Azambuja Amaral: Design of the work, data collection, data interpretation, drafting the article, critical revision of the article and final approval of the version to be published.

Eduarto Mundstock: Design of the work, data collection, data interpretation, drafting the article, critical revision of the article and final approval of the version to be published.

Camila H. Scarpatto: Drafting the article, critical revision of the article and final approval of the version to be published.

Wilson Cañon-Montañez: Data analysis and interpretation, drafting the article, critical revision of the article and final approval of the version to be published.

Rita Mattiello: Conception or design of the work, data collection, data analysis and interpretation, drafting the article, critical revision of the article and final approval of the version to be published.

## Declaration of Competing Interest

The authors declare no conflicts of interest.
